# Exploring the Relationship between Mood Disorders and Coexisting Health Conditions: The Focus on Nutraceuticals

**DOI:** 10.3390/brainsci13091262

**Published:** 2023-08-30

**Authors:** Agnieszka Mechlińska, Mariusz S. Wiglusz, Jakub Słupski, Adam Włodarczyk, Wiesław J. Cubała

**Affiliations:** Department of Psychiatry, Faculty of Medicine, Medical University of Gdańsk, Smoluchowskiego 17, 80-214 Gdańsk, Poland; mwiglusz@gumed.edu.pl (M.S.W.); jslupski@gumed.edu.pl (J.S.); adam.wlodarczyk@gumed.edu.pl (A.W.);

**Keywords:** major depressive disorder, bipolar disorder, nutraceuticals, comorbidity, nutritional support

## Abstract

Major depressive disorder and bipolar disorder are the leading causes of global disability. Approximately 50% of patients fail to attain remission, prompting a pronounced focus on the significance of dietary patterns and specific nutrients within the pathophysiology of mood disorders. The connection between chronic diseases and mood disorders follows a bidirectional pattern: physical ailments are interrelated with affective disorders, and, concurrently, mood symptoms often precede chronic diseases and have the potential to worsen their prognosis. Nutraceuticals affect factors that could potentially impact the onset of mood disorders: monoamines and brain-derived neurotrophic factor (BDNF) concentrations, neuroinflammation, oxidative stress, and sleep quality. Furthermore, mood disorders rarely manifest in isolation. Typically, such patients concurrently experience other mental disorders or somatic comorbidities: obesity, hypertension, diabetes, polycystic ovary syndrome (PCOS), etc., where providing nutritional support is also pertinent. To optimize the therapeutic approach for individuals with mood disorders, incorporating nutritional support may not solely ameliorate symptoms stemming directly from the mental condition, but also indirectly through interventions targeting comorbidities.

## 1. Introduction

In 2019, major depressive disorder (MDD) and bipolar disorder (BD) were experienced by, respectively, 280 million and 40 million people [1]. Mood disorders represent enduring and debilitating conditions characterized by shared pathophysiological mechanisms and clinical manifestations, but at least one manic/hypomanic episode should be present to diagnose BD [2]. Accurately distinguishing and diagnosing mood disorders remains a formidable task for medical practitioners; the early beginning of MDD, psychotic features, substance abuse, severe depression, and emergence of suicidal ideation should be considered as clinical indicators that may contribute to the prospective reclassification into the diagnostic category of BD [2].

MDD and BD are the leading causes of global disability; they affect daily functioning, decrease life quality, and increase mortality [3]. The therapeutic impact of most antidepressant agents stems from their interaction with monoamine neurotransmitters; additionally, these agents influence processes such as neurogenesis and neuromodulation. In managing manic episodes associated with BD, substances like lithium and atypical antipsychotics are often used [4,5]. Although many efficacious pharmacological agents are accessible, nearly 50% of patients remain unable to attain remission [4], and, with each successive episode, the likelihood of achieving remission diminishes [6].

Mood disorders are heterogeneous conditions of behavior, metabolism, and appetite aberrations. The significance of dietary patterns and distinct nutrients has been underscored in the pathophysiology of mood disorders [7,8]. Depressed individuals frequently experience deficiencies in dietary nutrients, and there seems to be a reciprocal relationship between depression and malnutrition [9]. Considering the potential benefits, interventions based on nutritional strategy as an adjunctive treatment appears promising for patients with mood disorders [10]. Nutraceuticals exhibit healing, neuroprotective, antioxidant, anti-inflammatory, and hypolipidemic properties [11]. Nutraceuticals are increasingly garnering research interest due to their established efficacy in managing chronic conditions, health-promoting capabilities, safety profile, and economic implications [12]. Due to their frequent utilization as adjunctive components in the treatment of mental disorders, The World Federation of Societies of Biological Psychiatry (WFSBP) and Canadian Network for Mood and Anxiety Treatments (CANMAT) have prepared guidelines for clinicians regarding their prescription, safety, and tolerability [13].

Within this manuscript, we present a hypothesis concerning a bidirectional effect between affective disorders and frequently cooccurring diseases, with particular attention on the influence of nutraceuticals, especially on the components contributing to the pathogenesis of these disorders.

## 2. Pathogenesis

**Figure 1:**
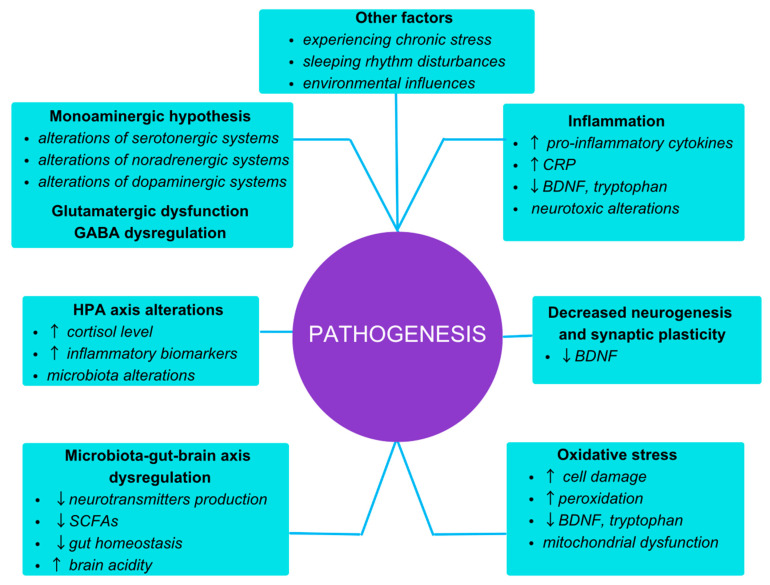
The factors influencing the pathogenesis of mood disorders [14,16,20,21,24,27,28,29,30,33,34,37,39,41,44,48,51,52,53].

Due to the heterogeneity of mood disorders, their etiology and pathophysiology are multifactorial. The treatment is challenging and necessitates a comprehensive assessment of the patient. Figure 1 summarizes the factors and mechanisms contributing to the onset of mood disorders.

Monoaminergic neurotransmission deficiency is one of the postulated theories of depression origin [14]. Serotonin, dopamine, and noradrenaline participate in brain activities, mood regulation, reward processing, and sleep. This explains why elevating monoamine concentrations via drugs like selective serotonin reuptake inhibitors (SSRIs), serotonin and noradrenaline reuptake inhibitors (SNRIs), and tricyclic antidepressants (TCAs) have antidepressant effects [15,16]. SSRIs and SNRIs are regarded as the initial choice of medication in recurrent depressive disorders’ pharmacological treatment [17]. SSRIs function by inhibiting the serotonin transporter (SERT), for which they have greater specificity than TCAs, resulting in serotonin level elevation within the synaptic cleft; additionally, SSRI are considered more tolerable when compared to other types of antidepressants [18,19]. If SSRIs do not produce the desired effect, SNRIs can contribute to the improvement of the patient’s condition due to their broader range of action, involving both serotoninergic and adrenergic systems [18]. In addition, neurotransmitters exhibit interdependence and exert an influence on their brain concentrations; modifying one of these neurotransmitters likely affects the functioning of others. Complementary to the monoamine hypothesis, glutamate and gamma-aminobutyric acid (GABA) systems are considered to be engaged in pathophysiology of MDD and BD [20]. The rapid therapeutic effect elicited by the administration of ketamine, an NMDA receptor antagonist, resulting in the alleviation of the inhibition of glutamate release in glutamate neurons, indicates the significance of the glutamate system in the pathogenesis of mood disorders [16].

Brain neuroplasticity and the growth, differentiation, and endurance of neurons constitute the primary functions attributed to BDNF. The influence of this neurotrophin on mood disorders has been investigated by numerous scientists. MDD and BD patients were observed to have decreased levels of BDNF [21], and certain medications have demonstrated potential efficacy by enhancing the improvement of BDNF functionality [22]. There is an association between serotonin and BDNF; the neurotransmitter is capable of stimulating the BDNF production, while the neurotrophin, in turn, augments serotonergic signaling [22]. The serotonin also participates in sleep–wake circadian cycle modulation. Simultaneously, BDNF’s involvement in insomnia and sleep deprivation has been indicated. Notably, in patients experiencing acute mood episodes of MDD and BD, BDNF levels are diminished, unlike in euthymic states. BDNF could potentially serve as a valuable biomarker for treatment, particularly in cases of MDD, as elevated levels are observed in individuals responding positively to therapeutic interventions, while non-responders exhibit no such alteration [23].

The growing body of evidence underscores the role of the immune system’s response in the underlying mechanisms of affective disorders. They exhibit a connection with chronic low-grade inflammation, characterized by elevated levels of pro-inflammatory biomarkers throughout their course [24]. An increase in the inflammatory immune response is associated with a state of neuronal impairment. Inflammatory stimulation leads to quantitative, functional, and morphological alterations in microglia. This gives rise to a reduction in the density of neurotransmitter, neurogenesis, and the quantity and dimensions of glial cells across diverse brain regions, such as the hippocampus, prefrontal cortex, amygdala, basal ganglia, and anterior cingulate [25]. Notably, SSRIs and SNRIs have demonstrated potential anti-inflammatory effects. Simultaneously, the current inflammatory state does not appear to interfere with their metabolism [26]. Osimo et al. [27] found that about 25% of patients with depression have CRP levels above 3 mg/L, which is considered a marker for low-grade inflammation; moreover, around 60% of patients have slightly elevated CRP levels (>1 mg/L). Psychological stress accompanying depression activates pro-inflammatory cytokines’ production. Among these cytokines, interleukin-6 (IL-6), tumor necrosis factor-alpha (TNF-alfa), and interleukin-1 (IL-1) hold particular significance in terms of their impact on brain function. Data suggest that IL-6, in particular, plays a pivotal role not only in pathogenesis of the disorder but also in its somatic manifestations and the response to antidepressant treatment. In addition, elevated levels of IL-6 contribute to the dysfunction in the hypothalamic–pituitary–adrenal (HPA) axis, synaptic neurotransmission alterations, and a reduction in neurotrophic factors [28]. It has been noted that, following the remission of symptoms associated with mood episodes, certain elevated pro-inflammatory cytokines can be restored [24]. In acute BD episodes, encompassing both manic and depressive phases, immune system gives rise to a pro-inflammatory response, inducing a concurrent reduction in BDNF [29]. While depressed patients with inflammatory disorders have demonstrated reduced tryptophan concentrations, a precursor for serotonin, it has been proposed that the immune system’s involvement in depression may occur through the kynurenine pathway of tryptophan. In the presence of inflammatory conditions, pro-inflammatory cytokines are produced, which induce tryptophan depletion by activating indoleamine 2,3-dioxygenase, converting tryptophan to kynurenine. Imbalances in the metabolites along the kynurenine pathway can potentially lead to neurotoxic alterations [30,31,32]. Patients with depression who also exhibit inflammation face a higher risk of developing physical health issues, particularly cardiovascular disease, and show reduced responsiveness to psychiatric interventions. Regular screening for inflammation, along with the identification and management of the underlying causes, has the potential to enhance overall health outcomes [27].

Oxidative stress is defined as a lack of balance between the generation of free radicals and the presence of endogenous antioxidants. In comparison to a healthy population, among individuals with MDD and BD, increased levels of oxidative stress were observed. This observation implies that oxidative processes, which can lead to cellular damage, might significantly influence the development and progression of mood disorders [33]. Oxidative stress increases the peroxidation of membrane lipids, proteins, and DNA, exerting a partial influence on neuroplasticity, uptake of neurotransmitters, and alterations in signaling pathways [34]. Moreover, scientific evidence indicates that disrupted mitochondrial homeostasis is a far-reaching feature in mood disorders, with both BD and MDD often being co-diagnosed with mitochondrial dysfunction. In the brain, mitochondria as energy generators are remarkably active. As very important organelles, they are also highly prone to damage caused by oxidative stress. In addition, the more serious mitochondrial dysfunction, the greater the oxidative stress causing neuronal damage [16]. Some medications used in mood disorder treatment, such as escitalopram, olanzapine, clozapine, lithium, and valproate, were identified to provide antioxidant activity, whereas antioxidant agents exhibited potentially positive effects in the realm of mood disorder treatment [35]. By modulating mitochondrial function, some accessible dietary supplements were evaluated as a potentially beneficial treatment, especially for BD [36].

The stress response is primarily regulated by the HPA axis, a key biological system. Cortisol, the principal outcome of this system, has a significant impact on cognitive and emotional processes in response to external stimuli. Moreover, it plays a role in shaping the development of the central nervous system (CNS) across an individual’s life. In individuals with Cushing’s disease, characterized by excessive cortisol production, symptoms encompassing depression, mania, and cognitive impairments are frequently observed. These symptoms can directly impact the function and structure of diverse regions within the CNS [37].

The dysregulated activity of the HPA axis has been linked to the pathogenesis of depression due to disruptions in the negative feedback mechanism, increased cortisol secretion, and inflammatory biomarkers. According to meta-analyses, morning cortisol rise precedes the later onset of MDD during adolescence. This observation implies that elevated cortisol may be a predictor rather than a consequence of depression [38]. Among patients with MDD, hypercortisolemia and inflammation have often been observed. These individuals are also more likely to develop glucocorticoid resistance [39]. In BD, HPA axis hyperactivity was noticed particularly in manic episodes [20]. Research in the neurobiology of depression has substantiated the crucial role played by the hippocampus as a structure proximal to hypothalamus within the HPA axis and possessing numerous corticosteroid receptors. The neurogenesis process, learning, and memory are the functions of the hippocampus which may be affected by stress in several ways. Diminished neuronal plasticity leads to HPA axis activation and elevation of corticosteroid levels [40].

Prolonged exposure to intense and enduring stress may be a cause of persistent physiological and psychological alterations throughout human development [41]. Early life stress (ELS) encompasses instances of physical, mental, and sexual abuse, including physical and emotional neglect during childhood. Other traumatic incidents, such as the loss of a primary caregiver, severe illness or injury, or natural disasters, also fall under the purview of ELS. Prolonged stress causes alterations in the noradrenergic system, which is interconnected with the neuroendocrine and immune systems [16]. ELS has been associated with various physical and mental disorders, including MDD. Interestingly, individuals who have experienced ELS have been found to exhibit elevated cortisol secretion levels, regardless of whether they have MDD or not. The HPA dysfunction that is observed in individuals with MDD appears to be more closely linked to the occurrence of ELS rather than solely the presence of MDD [42]. Psychosocial stress may affect blood pressure contributing to hypertension development—a primary driver of early cardiovascular and neurovascular disorders. Furthermore, individuals with anxiety and depression face a higher risk of hypertension [43].

Individual behavior may play a key role in mood disorder development. Cai et al. [44] identified sleep patterns such as insomnia and short sleep duration to be risk factors for BD. A meta-analysis focusing on sleep disturbances has revealed that the most substantial deviations in sleep continuity, REM sleep pressure, and sleep depth were linked to MDD [45]. Sleep disturbances may indicate the onset of mental disorders. A bidirectional relation exists between depression and sleep disturbances; depression can lead to insomnia, while insomnia can also precipitate the onset of depression [46,47].

While a positive correlation linking depression and conditions such as irritable bowel syndrome (IBS) has been established, the interconnections between the gut and the brain have garnered notable attention from the research community. The gut microbiota is defined as a complex collection of viruses, bacteria, archaea, protozoa, and fungi inhabiting the gastrointestinal tract in humans. The term microbiota–gut–brain axis describes the communication pathways between microbiota, the digestive tract, and the nervous system. Their channels of communication occur through neural, endocrine, and immune pathways [40]. Gut microbiota composition might be perturbed due to hyperactivation of the HPA axis [48]. These imbalances have been implicated in various mental diseases, exerting an impact on cognitive function, mood and emotion regulation, and interpersonal communication, potentially by intermediation via neuro-immune integration. Deterioration in both the richness and diversity of the gut microbiota has been observed among individuals with depression, attributed to elevated inflammation and cortisol levels [49].

The gut microbiota contributes to the production of neurotransmitters implicated in mental disorders. Lactobacillus and Bifidobacterium strains produce GABA, with Lactobacillus also being a source of acetylcholine. Escherichia, Saccharomyces, and Bacillus produce norepinephrine. Serotonin is produced by Streptococcus, Candida, Escherichia, and Enterococcus, while dopamine is attributed to Bacillus and Serratia [50]. In addition to their direct impact on neurotransmitters, microbiota also generate their precursors (e.g., tryptophan, tyrosine), suggesting an indirect influence on brain function [51]. Studies have demonstrated that probiotics, described as live microorganism beneficial for human health, when dosed in appropriate amounts possess the capacity to alleviate symptoms associated with depressive disorders, normalize the levels of corticosterone, noradrenaline, and BDNF, and improve immune functions as well [52]. In addition, the final products of microbiota metabolism—short-chain fatty acids (SCFAs)—have been observed to make an impact on the central nervous system. These effects encompass alterations in neurotransmitter production, modulation of lipid metabolism, influence on mitochondrial and immune functions, and modifications in DNA expression [53].

Insufficient data exist to elucidate the distinctions in gut microbiota between BD and other psychiatric conditions, as well as across various phases of BD [54]. Upon evaluating the composition of the intestinal microbiota, the following conclusions may be drawn: an abundance of some bacterial genera is similar between mental disorders—in particular, MDD and BD—as the same category can share more commonalities, but, at the same time, the composition can differ, especially compared to healthy controls. Enterococcus and Streptococcus, bacteria producing lactic acid, were found to be higher in both MDD and BD, while Escherichia/Shigella were higher in MDD and Bifidobacterium in BD. Given the observation of heightened acidity in the brains of patients with MDD and bipolar disorder BD, the potential involvement of lactate accumulation supported by lactic acid producers has been hypothesized in the context of pathophysiology [54]. A decline in the population of bacteria responsible for the production of butyrate has been noted in individuals affected by MDD, BD, and schizophrenia. This SCFA assumes a role in preserving gut homeostasis, upholding the integrity of the gut barrier, and influencing the immune system. The systemic inflammation observed in psychiatric disorders could potentially stem from diminished levels of butyrate-producing microorganisms. Moreover, certain bacteria associated with GABA metabolism were found to be heightened in cases of MDD and BD [54].

## 3. The Concept of Comorbidity and Its Implication for Disease Prognosis and Modifiable Factors

The connection between chronic diseases and mood disorders follows a bidirectional pattern: physical ailments are interrelated with affective disorders, and, concurrently, mood symptoms often precede chronic diseases and have the potential to worsen their prognosis [55].

In autoimmune diseases, inflammation can lead to an increased permeability of the blood–brain barrier. This heightened permeability facilitates the passage of cytokines and autoantibodies into the brain, thereby influencing the central nervous system. Consequently, this can give rise to a spectrum of psychiatric and neurological symptoms. Autoimmune conditions, for instance, thyroid diseases, rheumatoid arthritis, psoriasis, atopic dermatitis, and vitiligo, often co-occur with mood disorders, especially with BD [55,56]. The concomitance of autoimmune thyroiditis with MDD suggests that depression may be a disorder related to the immune system or may affect its functioning [57]. Interestingly, treatment-resistant depression demonstrates a greater prevalence of allergic and autoimmune diseases, alongside the presence of low-grade inflammatory biomarkers, compared to non-resistant MDD cases. Notably, chronic cardiovascular diseases are more likely to occur in individuals with low-grade inflammation [58]. Furthermore, metabolic syndrome, diabetes mellitus, and gout are health conditions associated with inflammation state [56]. Insulin resistance, neurohormonal activation, and chronic inflammation are the most significant factors in the onset, advancement, and transformation of metabolic syndrome into cardiovascular disease among all the suggested mechanisms [59].

Metabolic disorders, encompassing conditions such as obesity, hypercholesterolemia, hypertriglyceridemia, hypertension, and metabolic syndrome, demonstrate a noteworthy prevalence within the domain of affective disorders [55]. Metabolic syndrome is markedly more prevalent among individuals afflicted with mood disorders in comparison to the healthy population [60]. Obesity and mood disorders exhibit a dual relationship. Firstly, obesity is linked to a persistent, low-level inflammatory state. Secondly, individuals who are obese have a greater likelihood of experiencing mood disorders, and, conversely, those with mood disorders are at a higher risk of developing obesity-related metabolic issues like diabetes mellitus type 2. Insulin resistance plays a crucial role in the development of metabolic complications related to the obesity. It may increase the likelihood of depressive symptoms occurring and further compromise the prognosis of mood disorders. For individuals with diabetes, mood disorders may negatively impact their disease management. Hence, enhancing glucose metabolism can potentially aid in treating mood disorders [61]. Bipolar disorder due to disturbed eating behaviors, metabolic impairments, and obesity may contribute to a greater risk of cardiovascular diseases and mortality attributed to this cause. It is estimated that 6–57% individuals with cardiovascular disease experience depression. Both MDD and BD can manifest concomitantly with cardiovascular events [62,63]. In addition, higher cortisol levels observed in depressive patients can lead to carbohydrate metabolism disorders, especially insulin resistance [64]. Metabolic abnormalities occur either over the course or constitute risk factors of polycystic ovary syndrome (PCOS) development. In addition, hirsutism, excessive body weight, fertility disorders, and dermatological issues appearing in this disorder can negatively affect the mental condition of women [65].

Mood disorders are often associated with gastrointestinal ailments. These associations can manifest both as primary somatic disorders and as secondary occurrences. Examination of the incidence of depression has revealed a considerably higher probability of occurrence in individuals with coeliac disease in contrast to the control group; such conclusions were not drawn for BD [66]. Similarly, there is a greater risk of depression in individuals with inflammatory bowel disease, ulcerative colitis, and Crohn’s disease [67]. Regarding irritable bowel syndrome (IBS), the findings suggest that the presence of depressive moods doubles the susceptibility to its onset [68]. In addition, dysbiosis, which is linked to the progression of IBS, may also exert an impact on the manifestation of psychiatric disorders [69].

It should be noted that, other than somatic comorbidities, mood disorders are frequently diagnosed among patients suffering from other mental disorders, especially among individuals with eating disorders. Approximately 75% of individuals with MDD will develop another mental illness at some point in their lives [3]. Individuals grappling with eating disorders face a notably heightened risk of suicide, with a risk over five times greater than that observed in the general population. This risk is particularly elevated in those with comorbid psychiatric conditions, such as major depressive disorder and anxiety disorders. Longer duration of eating disorders’ symptoms probably leads to more severe symptoms of psychiatric comorbidities [70]. Binge-eating disorder (BED) and night-eating syndrome (NES) are linked to overweight, obesity, and medical comorbidities related to weight gain (metabolic syndrome components, diabetes) [71]. Binge eating has a deceptive effect: on the one hand, it is a stress-relieving method, but, on the other hand, it engenders mental distress. It has been estimated that 80% of BED individuals have experienced another mental disorder, particularly mood disorders.

## 4. Body Weight and Compliance with Medication

According to Semahegn et al. [72], a substantial proportion of patients grappling with MDD and BD demonstrated non-adherence to their prescribed medication regimen, with rates of 50% and 44%, respectively. Individuals’ behaviors—for instance, substance abuse, attitude towards medication, and social stigma—and clinical factors, such as medication side-effect and efficacy, comorbidity, and treatment duration, were emphasized by the authors. Weight gain was indicated as an adverse effect associated with non-adherence. Numerous antidepressants used as a first-line treatment contribute to weight alterations, whereas, in populations afflicted by psychiatric disorders, a higher prevalence of obesity has been observed. This condition is also linked with additional metabolic complexities, including diabetes mellitus and cardiovascular pathologies. Treatment duration plays a role as well; initial weight loss caused by SSRIs after the prolonged exhibition may be replaced by weight gain due to carbohydrate cravings [73]. In the event of a body weight change of ≥1.5 kg during the treatment, the medication is categorized as having a high risk of causing weight gain. The following medications fall under this category: citalopram, paroxetine, mirtazapine, amitriptyline (notriptyline), and phenelzine [73]. It remains challenging to balance the benefits of antidepressant treatment with adverse effects. Factors affecting patients’ non-compliance should be considered as necessary during the process of intervention design. This approach ensures that both patients and healthcare professionals can effectively attain the intended therapeutic objectives [72].

## 5. Nutraceuticals and Their Impact on Mood Disorders

The term “nutraceuticals” was first presented by Stephen L. Defelice in 1989 to describe food, or its components, providing benefits for human health, including disease prevention and treatment [74]. Providing a singular, specific definition for nutraceuticals continues to pose a challenge. This term, as a combination of words “nutrition” and “pharmaceuticals”, covers natural bioactive substances from edible sources, dietary supplements, probiotics and prebiotics, functional food, herbals, etc. [75]. Evidence suggests that nutraceuticals are emerging as promising adjunctive therapy for several chronic diseases. Due to neurobiological effects, neuroprotection and enhancing re-uptake of inhibited monoamines, it has been emphasized that they strengthen the effectiveness of medical therapy and alleviate side effects in managing mood disorders [11]. In accordance with the guidelines, omega-3 fatty acids, vitamin D, probiotics, N-acetyl cysteine, S-adenosyl-methionine, folate-based compounds, zinc, magnesium, vitamin C, tryptophan, creatine, and inositol deserve attention in the adjunctive treatment of mood disorders [13] (Table 1).

Numerous properties are attributed to omega-3 fatty acids, not only in psychiatric, but also across various somatic disorders. They modulate neurotransmission and neuronal function; they also manifest anti-inflammatory, anti-arteriosclerotic, and antithrombotic properties [76]. In addition, their administration could offer enhanced advantages in individuals with heightened inflammation, dietary insufficiency, or obesity [13]. Vitamin D exerts an influence on the synthesis of key neurotransmitters and neurons, while also possessing anti-inflammatory properties, potentially conferring supplementary advantages, particularly during the winter months [13,29,77,78]. Probiotics play a role in the creation and control of neurotransmitters and BDNF, influence the immune system and HPA axis, and enhance cognitive functions [79,80]. Zinc modulates synaptic plasticity and affects memory, emotional, and psychomotor functions [81]; it participates in neurotransmitter regulation and additionally exhibits antioxidant properties, which appear to hold significance and explains the effectiveness, especially in cases of compromised immunity and systemic inflammation [13,82]. Folates assume a pivotal role in the regulation of homocysteine levels, concurrently participating in transmethylation processes within the central nervous system and contributing to the metabolism of monoamine neurotransmitters [83]. In situations where conditions such as obesity, pregnancy, and inflammation coexist, the utilization of folates can offer supplementary advantages [13]. S-adenosyl-methionine is involved in neurotransmitter metabolism and methylation processes, which affect the fluidity and functioning of the neuronal membrane [83]. Vitamin C influences neurotransmitter concentration. It exhibits antioxidant, neurotrophic, and neuromodulator properties [84]. It can be particularly beneficial in conditions of oxidative stress and compromised immunity [13]. The most emphasized role of tryptophan is being a precursor for serotonin; it improves cognitive function and sleep quality [85]. Research has demonstrated that supplementing with creatine can enhance mitochondrial function and decrease susceptibility to apoptosis; its inadequate levels in the brain have been associated with neuronal dysfunction, mental retardation, brain atrophy, and depression [86,87]. Inositol has been shown to have a positive role on neurological and metabolic disease treatment; it is known as an effective insulin-sensitizer, and glial cell marker. In addition, it affects sleep quality [88]. Magnesium participates in glutamatergic transmission; it modulates neurotransmitters, BDNF levels, HPA axis, immune function, and sleep–wake cycle [89]. N-acetylcysteine, whose action can yield effects, especially during oxidative stress, provides cysteine for glutathione production, exhibits anti-inflammatory properties, and modulates neurotransmission [13,83].

**Table 1:** Analysis and summary of the effects of nutraceuticals utilized in mood disorders.

	**Major Depressive Disorder**	**Bipolar Disorder**
Vitamin D A sterol-derived nutritional compound, comprising a spectrum of 50 metabolites. Vitamin D is essential for calcium absorption in the gut and maintaining adequate calcium and phosphate concentrations in the blood. ○ The raw value indicates the level of vitamin D in the body, which can help determine bone health, immune system function, and overall vitamin D status [90].	Participating in the neurotransmitters synthesis Enhancing the immune system Enhancing neurogenesis	
	1500–4000 IU daily ^4^	Lack of data
	Expected to offer heightened benefits during the winter season [13].	
Omega-3 fatty acids Polyunsaturated fatty acids, including eicosapentaenoic acid (EPA) and docosahexaenoic acid (DHA), are indispensable nutritional compounds that have numerous health benefits, including reducing inflammation and supporting brain health. They are mainly acquired through dietary intake. ○ The raw value indicates the level of omega-3s in the body, which can provide insights into cardiovascular health and cognitive function [91].	Neurotransmission modulation Enhancing neurogenesis Enhancing immune system Preserving the integrity of the neuronal cell membrane	
	1–2 g of eicosapentaenoic acid ^1,8^	1–2 g of eicosapentaenoic acid ^3^
	There is a lack of evidence supporting a reduction in mania or hypomania; however, it may be still efficacious for individuals with elevated inflammation and/or obesity [13].	
Probiotics Live microorganisms which, while being administered in optimal doses, bestow a health benefit upon the host organism. ○ The raw value indicates the concentration and diversity of these bacteria, which can impact digestive health and immune function [52].	The production and control of neurotransmitters Enhancing cognitive functions Enhancing immune system Improving BDNF levels HPA axis modulation Reduction of pro-inflammatory bacteria Mitigating the adverse effect of antibiotic administration	
	1–10 billion units daily ^2,5^	Lack of data
	The optimal probiotic strains for treating depression have not been established [13].	
Zinc A trace mineral essential playing a pivotal role in numerous fundamental physiological functions; it is known as a cofactor in ≥300 enzymes. ○ Its raw value can provide insights into immune health, wound healing, and overall mineral status [81].	Neuroplasticity modulation Preserving the integrity of the neuronal cell membrane Enhancing memory and learning mechanisms Enhancing cognitive functions Increasing BDNF levels Participating in glutamatergic transmission Pro-inflammatory cytokine reduction	
	~25 mg hydroaspartate or sulphate ^2^	Lack of data
	May be beneficial for comorbid conditions involving weakened immunity, increased inflammation, or elevated oxidative stress, particularly in cases of dietary deficiency. Proven to be safe, but potential for causing nausea when taken on an empty stomach [13].	
Magnesium An indispensable mineral known as a cofactor in over 600 enzymes, it is engaged in CNS proper functioning and DNA reactions [83]. ○ Its raw value can indicate potential deficiencies that are relevant for nerve impulse transmission and muscle contraction, cardiovascular health, and overall neurological function [89].	Reducing neuronal hyperexcitability Increasing the availability of GABA Counteracting the inflammation Anxiety modulation Microbiota–gut–brain axis modulation	
	100–400 mg daily ^7^	Lack of data
	At elevated dosages, there exists a potential for interference with mineral absorption and their subsequent reduction; furthermore, such doses can also precipitate gastrointestinal disturbances [13].	
Vitamin C Vitamin C, also known as ascorbic acid, is an essential water-soluble micronutrient involved in tissue repair and the enzymatic production of certain neurotransmitters. ○ Its raw value reflects the body’s antioxidant levels and its ability to combat oxidative stress [92].	Participating in neuromodulation Participating in neurotransmitters transformation Counteracting the inflammation	
	~1 g/day ^7^	Lack of data
	May be beneficial for comorbid conditions involving weakened immunity or elevated oxidative stress. Proven to be safe, but potential for causing gastrointestinal upset [13].	
Folate-based compounds A vital compound important to the synthesis of methionine by conveying single-carbon units [lam]. It leads to a reduction in homocysteine levels and contributes to the production of monoamines [83]. ○ Its raw value can indicate potential deficiencies, which are especially significant during pregnancy due to its role in preventing neural tube defects [12].	Participating in neurotransmitters metabolism Engaging in the development of the nervous system Protection against neurotoxicity	
	15 mg of methylfolate ^2^	Lack of data
	Additional benefits can be obtained by addressing factors such as obesity, preconception care, pregnancy, and inflammation [13].	
S-Adenosyl Methionine It is a crucial compound for metabolic pathways, as it provides a methyl group that impacts gene expression regulation. When this regulation occurs improperly, it can lead to disturbances in the function of the nervous system. ○ Its raw value can provide insights into mental health and the body’s methylation processes [83].	Participating in the neurotransmitters synthesis Participating in maintaining membrane fluidity Neuroprotective properties Counteracting the inflammation	
	800 mg daily ^8^ 1600–3200 mg ^3^	There is a risk of triggering manic episodes.
N-Acetyl Cysteine A compound arises from L-cysteine subjected to acetylation, recognized for its role as a precursor to glutathione regarded as antioxidant. It is often used as a medication to treat acetaminophen overdose. ○ Its raw value can indicate antioxidant capacity and liver health [93].	Shielding against oxidative stress damage Pro-inflammatory cytokines reduction Neuroprotection Neurotransmitter modulation	
	Lack of data	1–3 g daily ^6^
	Additional benefits can be attained under conditions of increased oxidative stress [13].	
Tryptophan An indispensable amino acid known as a serotonin precursor, which can be delivered from protein-rich food sources. ○ The raw value can indicate the body’s ability to produce serotonin and can be linked to mood disorders if levels are imbalanced [85].	Participating in serotonin synthesis Sleep rhythm regulation Enhancing cognitive functions Enhancing the immune system	
	50–200 mg of 5-HTP/1 g of tryptophan ^7^	Lack of data
	A possibility of uncommon risk of serotonin syndrome [13].	
Creatine It is a guanidine compound synthesized by certain bodily organs involving the amino acids arginine and glycine in its production, and additionally requiring methionine as a donor of a methyl group. ○ Creatine is widely recognized for its significance in enhancing physical performance, muscle growth, and certain neurological functions [87].	Enhancing nervous system operation Protection against brain atrophy	
	5 g daily ^7^	Lack of data
	Renal disturbances should be carefully considered when contemplating administration [13].	
Inositol A polyol with myo-inositol as a predominant isomer, which can be obtained both from diet, especially fresh fruits and vegetables, and through endogenous synthesis. Inositol plays a role in various cellular processes, including cell growth and insulin signal transduction. ○ Its raw value can provide insights into metabolic health and cellular function [94].	Affecting sleep quality Participating in neurotransmission Marker of glial cells Improving insulin sensitivity	
	12 g daily ^7^	Lack of data
	Risk of occurring gastrointestinal disturbances [13].	

The table presents a summary of the effects exerted by nutraceuticals on MDD and BD, considering the preparations and dosages. Information regarding the recommended use as an adjunct or monotherapy has been included [13] (^1^ recommended as an adjunct; ^2^ temporarily recommended as an adjunct; ^3^ mildly recommended as an adjunct; ^4^ mildly recommended as an adjunct or monotherapy; ^5^ mildly recommended as a monotherapy; ^6^ presently not advised as an adjunct; ^7^ presently not advised as an adjunct or monotherapy; ^8^ presently not advised as a monotherapy). 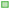
—the names of individual nutraceuticals along with supplementary information; 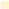
—potential involvement in the mood disorders mangement; 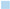
—suggested formulations and dosages; 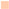
—additional information.

**Figure 2:**
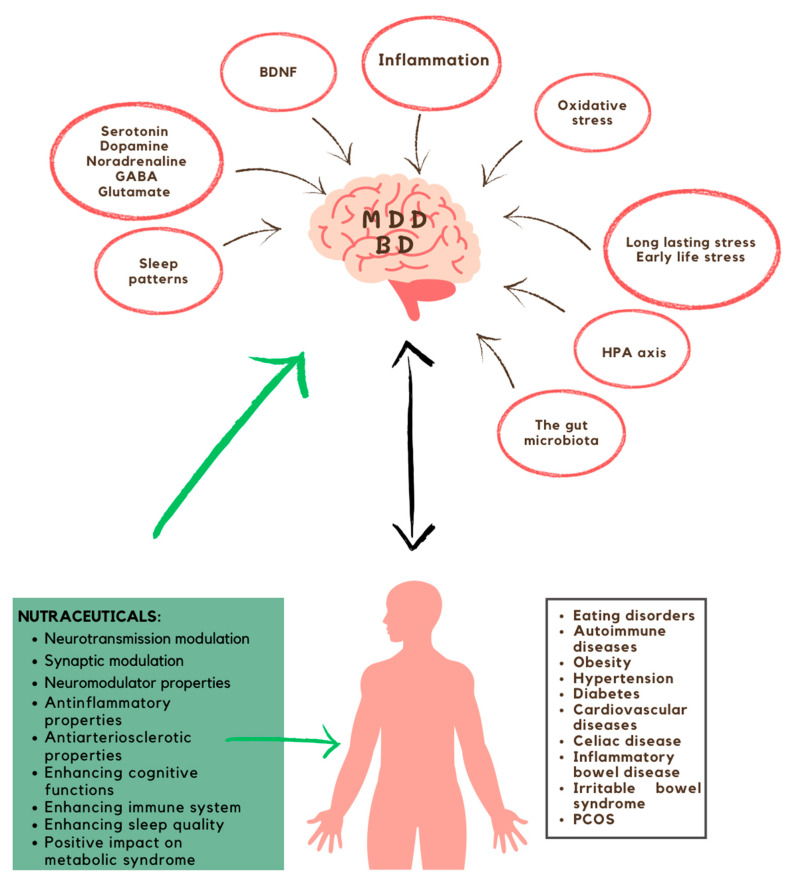
Summary of nutraceuticals’ impact on the mechanisms involved in the development of mood disorders. Nutraceuticals have the potential to directly target the underlying factors contributing to the onset and advancement of mood disorders, as well as indirectly through a positive effect on the somatic and autoimmune conditions frequently associated with major depressive disorder and bipolar disorder.

Omega-3 unsaturated fatty acids are acids that are endorsed as a pertinent component of a heart-healthy diet due to their capacity to mitigate the risk of cardiovascular diseases by acting positively on inflammation, vascular and endothelial function, and the composition of atherosclerotic plaques [95]. Eicosapentaenoic acid and docosahexaenoic acid appear to confer a protective benefit in diseases associated with metabolic syndromes [59]. Inositol, due to its insulin-sensitizing effect, seems to have a positive effect on the treatment of PCOS [96]. The utilization of probiotic and symbiotic supplements is a promising approach to managing metabolic syndrome in patients with prediabetes, with potential implications for preventing diseases linked to metabolic syndrome, such as type 2 diabetes, and other chronic conditions that significantly impact public health [97]. The oral administration of magnesium supplements can help to improve the occurrence of metabolic syndrome by reducing high blood pressure, hyperglycemia, and hypertriglyceridemia. This underscores the potential utility of oral magnesium supplementation as an adjunctive treatment for these occurrences, especially in individuals with low magnesium levels [98]. Consuming probiotics has been recommended to improve general health and immune function. It is currently acknowledged that the gut microbiota in humans might contribute to the emergence of metabolic disorders such as diabetes, obesity, and intestinal disorders including irritable bowel syndrome, inflammatory bowel disease, and coeliac disease. Significantly, research indicates that probiotic administration may enhance the treatment and perspectives for these conditions [99]. Dietary supplements appear to confer a favorable impact on enhancing sleep quality and daytime functioning [100] (Figure 2).

## 6. Conclusions

Research indicates a correlation between dietary exclusions and an elevated susceptibility to depressive symptoms. Notably, a higher number of exclusions correspond to an augmented risk. Furthermore, there exists a notion that the influence of depressive symptoms on dietary exclusions might not be as pronounced as the impact of exclusions on the manifestation of depressive symptoms [101]. The influence of nutrients on mood disorders underscores the interconnectedness between individuals’ mental well-being and their nutritional status, the quality of their diet, and adequate intake of essential vitamins and minerals [102].

Numerous variables influence the progression, course, and management of mood disorders. These variables encompass a diverse array of factors, and the identification of specific individual components that determine not only risk reduction for the disorder but also the efficacy of pharmacological interventions, particularly concerning long-term remission, remains intricate. To conclude, nutraceuticals exert an influence on factors that hold the potential to influence the initiation of mood disorders, encompassing concentrations of monoamines and BDNF, neuroinflammation, oxidative stress, and the quality of sleep. Furthermore, mood disorders rarely manifest in isolation. Typically, such patients concurrently experience other mental disorders or somatic comorbidities: obesity, hypertension, diabetes, PCOS, etc. It is noteworthy that the co-occurrence of immune system disorders with affective disorders is not uncommon, considering the presence of inflammatory processes and heightened indicators of such a state among individuals with MDD and BD. To optimize the therapeutic approach for individuals with mood disorders, incorporating nutritional support may not solely ameliorate symptoms stemming directly from the mental condition but also indirectly through interventions targeting comorbidities. Regarding implications for future research, it seems that real-world evidence supporting this concept would be of the interest for future studies.

## Data Availability

Not applicable.

## References

[1] World Health Organization. https://www.who.int/news-room/fact-sheets/detail/mental-disorders.

[2] Nestsiarovich A., Reps Jenna M., Matheny Michael E., DuVall S., Lynch K., Beaton M., Jiang X., Spotnitz M., Pfohl S.R., Shah N.H. (2021). Predictors of diagnostic transition from major depressive disorder to bipolar disorder: A retrospective observational network study. Transl. Psychiatr..

[3] Rakofsky J., Rapaport M. (2018). Mood Disorders. Continuum. Behav. Neurol. Psychiatr..

[4] Jones B.D.M., Daskalakis Z.J., Carvalho A.F., Strawbridge R., Young A.H., Mulsant B.H., Ishrat Husain M. (2020). Inflammation as a treatment target in mood disorders: Review. BJPsych Open.

[5] Sarno E., Moeser A.J., Robison A.J. (2021). Neuroimmunology of depression. Adv. Pharmacol..

[6] Wilkowska A., Cubała W.J. (2022). The Downstaging Concept in Treatment-Resistant Depression: Spotlight on Ketamine. Int. J. Mol. Sci..

[7] Koning E., Vorstman J., McIntyre R.S., Brietzke E. (2022). Characterizing eating behavioral phenotypes in mood disorders: A narrative review. Psychol. Med..

[8] Ashton M.M., Kavanagh B.E., Marx W., Berk M., Sarris J., Ng C.H., Hopwood M., Williams L.J., Dean O.M. (2021). A Systematic Review of Nutraceuticals for the Treatment of Bipolar Disorder. Can. J. Psychiatr..

[9] Ortega M.A., Fraile-Martínez O., García-Montero C., Alvarez-Mon M.A., Lahera G., Monserrat J., Llavero-Valero M., Gutiérrez-Rojas L., Molina R., Rodríguez-Jimenez R. (2022). Biological Role of Nutrients, Food and Dietary Patterns in the Prevention and Clinical Management of Major Depressive Disorder. Nutrients.

[10] Ceolin G., Breda V., Koning E., Meyyappan A.C., Gomes F.A., Moreira J.D., Gerchman F., Brietzke E. (2022). A Possible Antidepressive Effect of Dietary Interventions: Emergent Findings and Research Challenges. Curr. Treat. Options Psychiatr..

[11] Makkar R., Behl T., Bungau S., Zengin G., Mehta V., Kumar A., Uddin M.S., Ashraf G.M., Abdel-Daim M.M., Arora S. (2020). Nutraceuticals in neurological disorders. Int. J. Mol. Sci..

[12] AlAli M., Alqubaisy M., Aljaafari M.N., AlAli A.O., Baqais L., Molouki A., Abushelaibi A., Lai K., Lim S.E. (2021). Nutraceuticals: Transformation of conventional foods into health promoters/disease preventers and safety considerations. Molecules.

[13] Sarris J., Ravindran A., Yatham L.N., Marx W., Rucklidge J.J., McIntyre R.S., Akhondzadeh S., Benedetti F., Caneo C., Cramer H. (2022). Clinician guidelines for the treatment of psychiatric disorders with nutraceuticals and phytoceuticals: The World Federation of Societies of Biological Psychiatry (WFSBP) and Canadian Network for Mood and Anxiety Treatments (CANMAT) Taskforce. World J. Biol. Psychiatr..

[14] Perez-Caballero L., Torres-Sanchez S., Romero-López-Alberca C., González-Saiz F., Mico J.A., Berrocoso E. (2019). Monoaminergic system and depression. Cell Tissue Res..

[15] Oakes P., Loukas M., Oskouian R.J., Tubbs R.S. (2017). The neuroanatomy of depression: A review. Clin. Anat..

[16] Dean J., Keshavan M. (2017). The neurobiology of depression: An integrated view. Asian, J. Psychiatr..

[17] Kennedy S.H., Lam R.W., McIntyre R.S., Tourjman S.V., Bhat V., Blier P., Hasnain M., Jollant F., Levitt A.J., MacQueen G.M. (2016). Canadian Network for Mood and Anxiety Treatments (CANMAT) 2016 ClinicalGuidelines for the Management of Adults with Major Depressive Disorder: Section 3. Pharmacological Treatments. Can. J. Psychiatr..

[18] Buzuk G.A., Owiecki M. (2014). Molecular mechanisms of anti-depressive drugs activity. Adv. Cell Biol..

[19] Edinoff A.N., Haseeb A.A., Hanna T.A., Ochoa C.O., Patti S.J., Ghaffar Y.A., Kaye A.D., Viswanath O., Urits I., Boyer A.G. (2021). Selective serotonin reuptake inhibitors and adverse effects: A narrative review. Neurol. Int..

[20] Sigitova E., Fišar Z., Hroudová J., Cikánková T., Raboch J. (2017). Biological hypotheses and biomarkers of bipolar disorder. Psychiatr. Clin. Neurosci..

[21] Lin C., Huang T. (2020). Brain-derived neurotrophic factor and mental disorders. Biomed. J..

[22] Schmitt K., Holsboer-Trachsler E., Eckert A. (2016). BDNF in sleep, insomnia, and sleep deprivation. Ann. Med..

[23] Polyakova M., Stuke K., Schuemberg K., Mueller K., Schoenknecht P., Schroeter M.L. (2015). BDNF as a biomarker for successful treatment of mood disorders: A systematic & quantitative meta-analysis. J. Affect. Disord..

[24] Bauer M.E., Teixeira A.L. (2021). Neuroinflammation in Mood Disorders: Role of Regulatory Immune Cells. Neuroimmunomodulation.

[25] Ruiz N.A.L., Del Ángel D.S., Brizuela N.O., Peraza A.V., Olguín H.J., Soto M.P., Guzmán D.C. (2022). Inflammatory Process and Immune System in Major Depressive Disorder. Int. J. Neuropsychopharmacol..

[26] Gałecki P., Mossakowska-Wójcik J., Talarowska M. (2018). The anti-inflammatory mechanism of antidepressants—SSRIs, SNRIs. Prog. Neuro-Psychopharmacol. Biol. Psychiatr..

[27] Osimo E.F., Baxter L.J., Lewis G., Jones P.B., Khandaker G.M. (2019). Prevalence of low-grade inflammation in depression: A systematic review and meta-analysis of CRP levels. Psychol. Med..

[28] Ting E.Y.C., Yang A.C., Tsai S.J. (2020). Role of interleukin-6 in depressive disorder. Int. J. Mol. Sci..

[29] Cereda G., Enrico P., Ciappolino V., Delvecchio G., Brambilla P. (2020). The role of vitamin D in bipolar disorder: Epidemiology and influence on disease activity. J. Affect. Disord..

[30] Won E., Kim Y. (2016). Stress, the Autonomic Nervous System, and the Immune-kynurenine Pathway in the Etiology of Depression. Curr. Neuropharmacol..

[31] Hebbrecht K., Skorobogatov K., Giltay E.J., Coppens V., De Picker L., Morrens M. (2021). Tryptophan Catabolites in Bipolar Disorder: A Meta-Analysis. Front. Immunol..

[32] Kadriu B., Farmer C.A., Yuan P., Park L.T., Deng Z.-D., Moaddel R., Henter I.D., Shovestul B., Ballard E.D., Kraus C. (2021). The kynurenine pathway and bipolar disorder: Intersection of the monoaminergic and glutamatergic systems and immune response. Mol. Psychiatr..

[33] Eriksen J.K.D., Coello K., Stanislaus S., Kjærstad H.L., Sletved K.S.O., McIntyre R.S., Faurholt-Jepsen M., Miskowiak K.K., Poulsen H.E., Kessing L.V. (2022). Associations between childhood maltreatment and oxidative nucleoside damage in affective disorders. Eur. Psychiatr..

[34] Madireddy S., Madireddy S. (2022). Therapeutic Interventions to Mitigate Mitochondrial Dysfunction and Oxidative Stress–Induced Damage in Patients with Bipolar Disorder. Int. J. Mol. Sci..

[35] Zheng W., Zhang Q., Cai D., Yang X.-H., Qui Y., Ungvari G.S., Ng C.H., Berk M., Ning Y.-P., Xiang Y.-T. (2018). N-acetylcysteine for major mental disorders: A systematic review and meta-analysis of randomized controlled trials. Acta Psychiatr. Scand..

[36] Toniolo R.A., de Brito Ferreira Fernandes F., Silva M., da Silva Dias R., Lafer B. (2016). Cognitive effects of creatine monohydrate adjunctive therapy in patients with bipolar depression: Results from a randomized, double-blind, placebo-controlled trial. J. Affect. Disord..

[37] Belvederi Murri M., Prestia D., Mondelli V., Pariante C., Patti S., Olivieri B., Arzani C., Masotti M., Respino M., Antonioli M. (2015). The HPA axis in bipolar disorder: Systematic review and meta-analysis. Psychoneuroendocrinology.

[38] Zajkowska Z., Gullett N., Walsh A., Zonca V., Pedersen G.A., Souza L., Kieling C., Fisher H.L., Kohort B.A., Mondelli V. (2021). Cortisol and development of depression in adolescence and young adulthood—A systematic review and meta-analysis. Psychoneuroendocrinology.

[39] Nikkheslat N., McLaughlin A., Hastings C., Zajkowska Z., Nettis M.A., Mariani N., Enache D., Lombardo G., Pointon L., Cowen P.J. (2019). Childhood trauma, HPA axis activity and antidepressant response in patients with depression. Brain. Behav. Immun..

[40] Mikulska J., Juszczyk G., Gawrońska-Grzywacz M., Herbet M. (2021). Hpa axis in the pathomechanism of depression and schizophrenia: New therapeutic strategies based on its participation. Brain Sci..

[41] Murphy F., Nasa A., Cullinane D., Raajakesary K., Gazzaz A., Sooknarine V., Haines M., Roman E., Kelly L., O’Neill A. (2022). Childhood Trauma, the HPA Axis and Psychiatric Illnesses: A Targeted Literature Synthesis. Front. Psychiatr..

[42] Ceruso A., Martínez-Cengotitabengoa M., Peters-Corbett A., Diaz-Gutierrez M.J., Martínez-Cengotitabengoa M. (2020). Alterations of the HPA Axis Observed in Patients with Major Depressive Disorder and Their Relation to Early Life Stress: A Systematic Review. Neuropsychobiology.

[43] Liu M., Li N., Li W.A., Khan H. (2017). Association between psychosocial stress and hypertension: A systematic review and meta-analysis. Neurol. Res..

[44] Cai J., Wei Z., Chen M., He L., Wang H., Li M., Peng Y. (2022). Socioeconomic status, individual behaviors and risk for mental disorders: A Mendelian randomization study. Eur. Psychiatr..

[45] Baglioni C., Nanovska S., Regen W., Spiegelhalder K., Feige B., Nissen C., Reynolds C.F., Riemann D. (2016). Sleep and mental disorders: A meta-analysis of polysomnographic research. Psychol. Bull..

[46] Hepsomali P., Groeger J.A. (2021). Diet, sleep, and mental health: Insights from the UK biobank study. Nutrients.

[47] Fang H., Tu S., Sheng J., Shao A. (2019). Depression in sleep disturbance: A review on a bidirectional relationship, mechanisms and treatment. J. Cell. Mol. Med..

[48] Mörkl S., Butler M.I., Holl A., Cryan J.F., Dinan T.G. (2020). Probiotics and the Microbiota-Gut-Brain Axis: Focus on Psychiatry. Curr. Nutr. Rep..

[49] Wieërs G., Belkhir L., Enaud R., Leclercq S., Philippart de Foy J.-M., Dequenne I., de Timary P., Cani P.D. (2020). How Probiotics Affect the Microbiota. Front. Cell. Infect. Microbiol..

[50] Gondalia S., Parkinson L., Stough C., Scholey A. (2019). Gut microbiota and bipolar disorder: A review of mechanisms and potential targets for adjunctive therapy. Psychopharmacology.

[51] Chen Y., Xu J., Chen Y. (2021). Regulation of neurotransmitters by the gut microbiota and effects on cognition in neurological disorders. Nutrients.

[52] Generoso J.S., Giridharan V.V., Lee J., Macedo D., Barichello T. (2021). The role of the microbiota-gut-brain axis in neuropsychiatric disorders. Braz. J. Psychiatr..

[53] Mirzaei R., Bouzari B., Hosseini-Fard S.R., Mazaheri M., Ahmadyousefi Y., Abdi M., Jalalifar S., Karimitabar Z., Teimoori A., Keyvani H. (2021). Role of microbiota-derived short-chain fatty acids in nervous system disorders. Biomed. Pharmacother..

[54] McGuinness A.J., Davis J.A., Loughman A., Collier F., O’Hely M., Simpson C.A., Greem J., Marx W., Hair C., Jacka F.N. (2022). A systematic review of gut microbiota composition in observational studies of major depressive disorder, bipolar disorder and schizophrenia. Mol. Psychiatr..

[55] Perugi G., Quaranta G., Belletti S., Casalini F., Mosti N., Toni C., Dell’Osso L. (2014). General medical conditions in 347 bipolar disorder patients: Clinical correlates of metabolic and autoimmune-allergic diseases. J. Affect. Disord..

[56] Ahuja M., Sathiyaseelan T., Wani R.J., Fernandopulle P. (2020). Obesity, food insecurity, and depression among females. Arch. Public. Heal..

[57] Karakatsoulis G.N., Tsapakis E.M., Mitkani C., Fountoulakis K.N. (2021). Subclinical thyroid dysfunction and major depressive disorder. Hormones.

[58] Lauden A., Geishin A., Merzon E., Korobeinikov A., Green I., Golan-Cohen A., Vinker S., Manor I., Weizman A., Magen E. (2021). Higher rates of allergies, autoimmune diseases and low-grade inflammation markers in treatment-resistant major depression. Brain Behav. Immun. Heal..

[59] Rochlani Y., Pothineni N.V., Kovelamusi S., Metha J.L. (2018). Metabolic syndrome: Pathophysiology, management, and modulation by natural compounds. Ther. Adv. Vaccines.

[60] Vancampfort D., Stubbs B., Mitchell A.J., De Hert M., Wampers M., Ward P.B., Rosenbaum S., Correll C.U. (2015). Risk of metabolic syndrome and its components in people with schizophrenia and related psychotic disorders, bipolar disorder and major depressive disorder: A systematic review and meta-analysis. World Psychiatr..

[61] Martins L.B., Tibães J.R.B., Berk M., Teixeira A.L. (2021). Diabetes and mood disorders: Shared mechanisms and therapeutic opportunities. Int. J. Psychiatr. Clin. Pract..

[62] Gardea-Resendez M., Winham S.J., Romo-Nava F., Cuellar-Barboza A., Clark M.M., Andreazza A.C., Cabello-Arreola A., Veldic M., Bondi D.J., Singh B. (2022). Quantification of diet quality utilizing the rapid eating assessment for participants-shortened version in bipolar disorder: Implications for prospective depression and cardiometabolic studies. J. Affect. Disord..

[63] Joelson A.M., Geller M.G., Zylberberg H.M., Green P.H.R., Lebwohl B. (2018). The effect of depressive symptoms on the association between gluten-free diet adherence and symptoms in celiac disease: Analysis of a patient powered research network. Nutrients.

[64] Joshua J., Golden J., Golden S.H. (2017). Cortisol dysregulation: The bidirectional link between stress, depression, and type 2 diabetes mellitus. Ann. N. Y. Acad. Sci..

[65] Cantelmi T., Lambiase E., Unfer V.R., Gambioli R., Unfer V. (2021). Inositol treatment for psychological symptoms in Polycystic Ovary Syndrome women. Eur. Rev. Med. Pharmacol. Sci..

[66] Clappison E., Hadjivassiliou M., Zis P. (2020). Psychiatric manifestations of coeliac disease, a systematic review and meta-analysis. Nutrients.

[67] Navabi S., Gorrepati V.S., Yadav S., Chintanaboina J., Maher S., Demuth P., Stern B., Stuart A., Tinsley A., Clarke K. (2018). Influences and impact of Anxiety and Depression in the setting of inflammatory bowel disease. Inflamm. Bowel Dis..

[68] Sibelli A., Chalder T., Everitt H., Workman P., Windgassen S., Moss-Morris R. (2016). A systematic review with meta-analysis of the role of anxiety and depression in irritable bowel syndrome onset. Psychol. Med..

[69] Pinto-Sanchez M.I., Hall G.B., Ghajar K., Nardelli A., Bolino C., Lau J.T., Martin F.-P., Cominetti O., Welsh C., Rieder A. (2017). Probiotic Bifidobacterium longum NCC3001 Reduces Depression Scores and Alters Brain Activity: A Pilot Study in Patients with Irritable Bowel Syndrome. Gastroenterology.

[70] Lin J.A., Jhe G., Vitagliano J.A., Milliren C.E., Spigel R., Woods E.R., Forman S.F., Richmond T.K. (2021). The Association of Malnutrition, illness duration, and pre-morbid weight status with anxiety and depression symptoms in adolescents and young adults with restrictive eating disorders: A cross-sectional study. J. Eat. Disord..

[71] McCuen-Wurst C., Ruggieri M., Allison K.C. (2018). Disordered eating and obesity: Associations between binge eating-disorder, night-eating syndrome, and weight-related co- morbidities. Ann. N. Y. Acad. Sci..

[72] Semahegn A., Torpey K., Manu A., Assefa N., Tesfaye G., Ankomah A. (2020). Psychotropic medication non-adherence and its associated factors among patients with major psychiatric disorders: A systematic review and meta-analysis. Syst. Rev..

[73] Gill H., Gill B., El-Halabi S., Chen-Li D., Lipsitz O., Rosenblat J.D., Van Rheenen T.E., Rodrigues N.B., Mansur R.B., Majeed A. (2020). Antidepressant Medications and Weight Change: A Narrative Review. Obesity.

[74] Aronson J.K. (2016). Defining ‘nutraceuticals’: Neither nutritious nor pharmaceutical. Br. J. Clin. Pharmacol..

[75] Ruchi S., Amanjot K., Sourav T., Keerti B., Sujit B. (2017). Role of nutraceuticals in health care: A review. Int. J. Green. Pharm..

[76] da Conceição Silva Chaves R., Aguiar O.B., Moreno A.B., Brunoni A.R., Molina M.d.C.B., Viana M.C., Bensoñor I., Griep R.H., da Fonseca M.D.J.M. (2022). Consumption of Omega-3 and Maintenance and Incidence of Depressive Episodes: The ELSA-Brasil Study. Nutrients.

[77] Parker G.B., Brotchie H., Graham R.K. (2017). Vitamin D and depression. J. Affect. Disord..

[78] Steardo L., Luciano M., Sampogna G., Carbone E.A., Caivano V., Di Cerbo A., Giallonardo V., Palummo C., Vece A., Fiorillo A. (2020). Clinical severity and calcium metabolism in patients with bipolar disorder. Brain Sci..

[79] Thangaleela S., Sivamaruthi B.S., Kesika P., Chaiyasut C. (2022). Role of Probiotics and Diet in the Management of Neurological Diseases and Mood States: A Review. Microorganisms.

[80] Del Toro-Barbosa M., Hurtado-Romero A., Garcia-Amezquita L.E., García-Cayuela T. (2020). Psychobiotics: Mechanisms of action, evaluation methods and effectiveness in applications with food products. Nutrients.

[81] Styczeń K., Sowa-Kućma M., Siwek M., Dudek D., Reczyński W., Szewczyk B., Misztak P., Topór-Mądry R., Opoka W., Nowak G. (2016). The serum zinc concentration as a potential biological marker in patients with major depressive disorder. Metab. Brain Dis..

[82] Jafari F., Mohammadi H., Amani R. (2021). The effect of zinc supplementation on brain derived neurotrophic factor: A meta-analysis. J. Trace Elem. Med. Biol..

[83] Hoepner C.T., Mcintyre R.S., Papakostas G.I. (2021). Impact of Supplementation and Nutritional Interventions on Pathogenic Processes of Mood Disorders: A Review of the Evidence. Nutrients.

[84] Bansal V., Chatterjee I. (2022). Association of Vitamins and Neurotransmitters: Understanding the Effect on Schizophrenia. Neurochem. J..

[85] Jenkins T.A., Nguyen J.C.D., Polglaze K.E., Bertrand P.P. (2016). Influence of tryptophan and serotonin on mood and cognition with a possible role of the gut-brain axis. Nutrients.

[86] Tan H.Z., Li H., Liu C., Guan J.-T., Guo X.-B., Wen C.-H., Ou S.-M., Zhang Y.-N., Zhang J., Xu C.-T. (2016). Main Effects of Diagnoses, Brain Regions, and their Interaction Effects for Cerebral Metabolites in Bipolar and Unipolar Depressive Disorders. Sci. Rep..

[87] Beal M.F. (2011). Neuroprotective effects of creatine. Amino Acids.

[88] Mashayekh-Amiri S., Delavar M.A., Bakouei F., Faramarzi M., Esmaeilzadeh S. (2022). The impact of myo-inositol supplementation on sleep quality in pregnant women: A randomized, double-blind, placebo-controlled study. J. Matern. Neonatal Med..

[89] Botturi A., Ciappolino V., Delvecchio G., Boscutti A., Viscardi B., Brambilla P. (2020). The role and the effect of magnesium in mental disorders: A systematic review. Nutrients.

[90] Alonso N., Zelzer S., Eibinger G., Herrmann M. (2023). Vitamin D Metabolites: Analytical Challenges and Clinical Relevance. Calcif. Tissue Int..

[91] Lange K.W. (2020). Omega-3 fatty acids and mental health. Glob. Health J..

[92] Wong S., Chin K., Ima-Nirwana S. (2020). Vitamin C: A Review on its Role in the Management of Metabolic Syndrome. Int. J. Med. Sci..

[93] Guerini M., Condro G., Friuli V., Maggi L., Perugini P. (2022). N-acetylcysteine (NAC) and Its Role in Clinical Practice Management of Cystic Fibrosis (CF): A Review. Pharmaceuticals.

[94] Croze M.L., Soulage C.O. (2013). Potential role and therapeutic interests of myo-inositol in metabolic diseases. Biochimie.

[95] Weinberg R.L., Brook R.D., Rubenfire M., Eagle K.A. (2021). Cardiovascular Impact of Nutritional Supplementation with Omega-3 Fatty Acids: JACC Focus Seminar. J. Am. Coll. Cardiol..

[96] Zhao H., Xing C., Zhang J., He B. (2021). Comparative efficacy of oral insulin sensitizers metformin, thiazolidinediones, inositol, and berberine in improving endocrine and metabolic profiles in women with PCOS: A network meta-analysis. Reprod. Health.

[97] Kassaian N., Feizi A., Aminorroaya A., Amini M. (2019). Probiotic and synbiotic supplementation could improve metabolic syndrome in prediabetic adults: A randomized controlled trial. Diabetes Metab. Syndr. Clin. Res. Rev..

[98] Rodríguez-Morán M., Simental-Mendía L.E., Gamboa-Gómez C.I., Guerrero-Romero F. (2018). Oral Magnesium Supplementation and Metabolic Syndrome: A Randomized Double-Blind Placebo-Controlled Clinical Trial. Adv. Chronic Kidney Dis..

[99] Kim S., Robin B.G., Kim Y., Kwon J., Kim H., Cho J.H., Kim H.B., Lee J.-H. (2019). Role of probiotics in human gut microbiome-associated diseases. J. Microbiol. Biotechnol..

[100] Wu Y., Huang X., Zhong C., Wu T., Sun D., Wang R., Zhan Q., Luo H. (2022). Efficacy of Dietary Supplements on Sleep Quality and Daytime Function of Shift Workers: A Systematic Review and Meta-Analysis. Front. Nutr..

[101] Matta J., Hoertel N., Airagnes G., Czernichow S., Kesse-Guyot E., Limosin F., Goldberg M., Zins M., Lemogne C. (2020). Dietary restrictions and depressive symptoms: Longitudinal results from the constances cohort. Nutrients.

[102] Ekinci G.N., Sanlier N. (2022). The relationship between nutrition and depression in the life process: A mini-review. Exp. Gerontol..

